# Supplementary material for: Antifungal Properties of Bio-AgNPs against D. pinodes and F. avenaceum Infection of Pea (Pisum sativum L.) Seedlings
Source: Int J Mol Sci. 2024 Apr 20;25(8):4525. doi: 10.3390/ijms25084525 (PMC11050071; doi:10.3390/ijms25084525)
Supplement: Supplementary file 1 [file ijms-25-04525-s001.zip › ijms-2929276-supplementary.pdf]

Table S1. Cotyledons fresh weight (FW) and dry weight (DW) of shoots, roots and cotyledons of 22-days-old pea seedlings, developed without infection (control), infected (on the 8th day of germination, DG) with *D. pinodes* or *F. avenaceum* after pre-treatment with water, fungicide and bio-AgNPs (at 100 and 200 mg/L).

			Control	Seedlings infected after short-term immersion in			
				Water	Fungicide*	bio-AgNPs	
						100 mg/L	200 mg/L
D. pinodes infection	FW (mg)	cotyledons	302.3[a]	275.8[a]	307.2[a]	276.5[a]	291.9[a]
	DW (mg)	shoots	34.6[a]	29.5[a]	30.3[a]	34.8[a]	32.3[a]
		roots	14.8[a]	13.1[a]	15.0[a]	16.0[a]	15.5[a]
		cotyledons	73.6[a]	74.9[a]	76.5[a]	70.9[a]	69.4[a]
F. avenaceum infection	FW (mg)	cotyledons	261.4[ab]	219.7[b]	255.3[ab]	109.9[a]	264.7[ab]
	DW (mg)	shoots	47.9[a]	38.9[a]	45.7[a]	46.3[a]	46.5[a]
		roots	24.8[a]	26.7[a]	22.3[a]	24.4[a]	21.6[a]
		cotyledons	57.9[a]	45.0[a]	58.4[a]	71.4[a]	59.5[a]

Means of 3 replicates. The same letters (a-b) by the values indicate statistically insignificant ($P<0.05$) differences (valid for roots, shoots and cotyledon separately) based on ANOVA analysis and Tukey's post-hoc corrections.

* The fungicide Amistar 250 SC (22.82% of azoxystrobin) was used against *D. pinodes* and Toledo Extra 430 SC (33.29% of tebuconazole) against *F. avenaceum*.

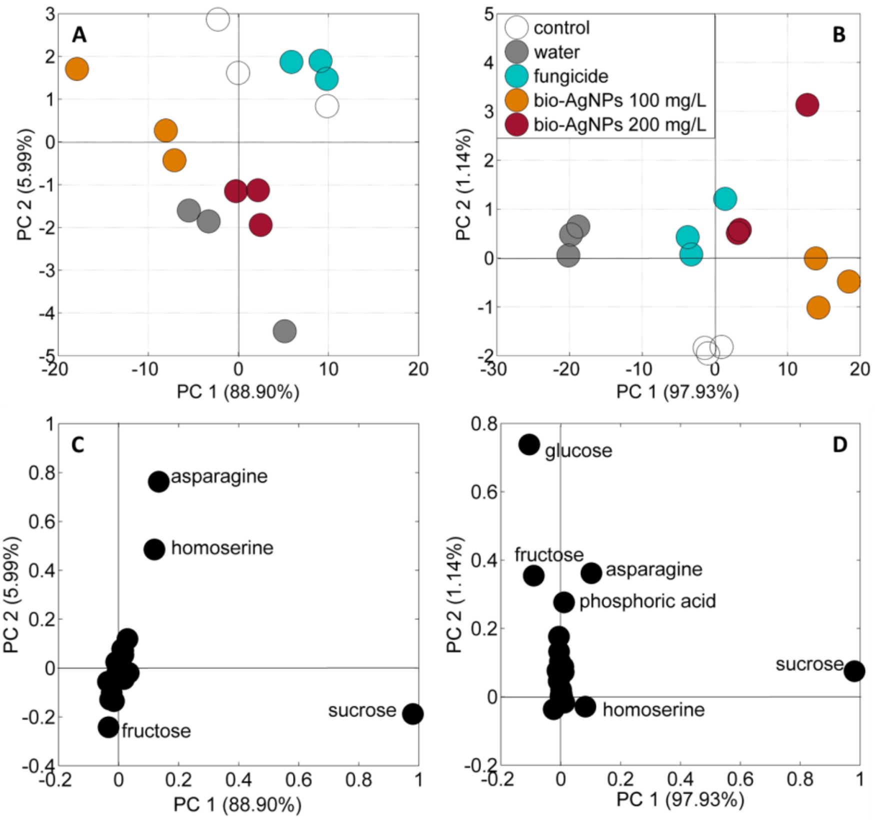

Figure S1. PCA (A, B) and loading plots (C, D) of cotyledon's metabolic profiles of 22-day-old seedlings of pea (*Pisum sativum* L.), 14 days after *D. pinodes* (A, C) and *F. avenaceum* (B, D) inoculation, respectively. Abbreviations: control – non-infected seedlings; water, fungicide, bio-AgNPs 100 and 200 mg/L – seedlings pretreated with water, fungicide (azoxystrobin or tebuconazole, respectively) or bio-AgNPs (at 100 and 200 mg/L), respectively, before infection with *D. pinodes* or *F. avenaceum*.

Table S2. The concentration of total identified polar metabolites (TIPMs), including total soluble carbohydrates (TSCs), total amino acids (TAAs), total organic acids (TOAs), and total remaining compounds (TRCs) **in roots** of 22-day-old pea seedlings (*Pisum sativum* L.), 14 days post-inoculation with *D. pinodes*.

	Control	*D. pinodes* infection			
		Water	Fungicide	bio-Ag NPs	
				100 mg/L	200 mg/L
**Metabolites**	mg/g DW				
**TIPMs**, including:	64.90^{b}	65.53^{b}	74.17^{a}	58.12^{c}	56.21^{c}
**TSCs**, including:	17.16^{ab}	15.74^{c}	17.70^{a}	16.83^{b}	12.29^{d}
fructose	0.05^{c}	0.10^{a}	0.06^{b}	0.06^{bc}	0.05^{c}
galactose	0.34^{a}	0.12^{d}	0.20^{c}	0.26^{b}	0.19^{c}
glucose	1.43^{a}	0.53^{e}	0.68^{d}	1.06^{b}	0.88^{c}
myo-inositol	1.25^{a}	0.99^{c}	0.97^{c}	1.16^{b}	0.90^{d}
sucrose	13.82^{b}	13.64^{b}	15.48^{a}	13.91^{b}	9.99^{c}
gluconic acid	0.28^{b}	0.37^{a}	0.31^{b}	0.38^{a}	0.28^{b}
**TAAs**, including:	36.19^{bc}	39.06^{b}	45.25^{a}	31.33^{c}	34.11^{bc}
alanine	0.40^{c}	0.60^{a}	0.59^{a}	0.34^{c}	0.51^{b}
asparagine	1.35^{c}	4.32^{a}	4.63^{a}	1.83^{bc}	2.59^{b}
aspartic acid	0.58^{b}	0.61^{ab}	0.64^{a}	0.50^{c}	0.49^{c}
β-alanine	0.02^{d}	0.08^{a}	0.05^{b}	0.04^{c}	0.04^{c}
GABA	0.34^{e}	3.28^{a}	2.15^{b}	1.71^{c}	1.54^{d}
glutamic acid	0.34^{d}	0.55^{b}	0.66^{a}	0.35^{d}	0.43^{c}
homoserine	30.94^{ab}	26.55^{bc}	33.75^{a}	24.40^{c}	26.33^{bc}
hydroxyproline	0.32^{a}	0.25^{b}	0.32^{a}	0.30^{ab}	0.27^{b}
isoleucine	0.29^{a}	0.31^{a}	0.28^{a}	0.28^{a}	0.27^{a}
lysine	0.00^{c}	0.13^{a}	0.07^{ab}	0.05^{bc}	0.09^{ab}
phenylalanine	0.15^{a}	0.17^{a}	0.18^{a}	0.14^{a}	0.20^{a}
proline	0.05^{c}	0.39^{a}	0.16^{b}	0.09^{c}	0.06^{c}
serine	0.48^{b}	0.63^{a}	0.64^{a}	0.49^{b}	0.41^{b}
threonine	0.64^{ab}	0.79^{a}	0.79^{a}	0.47^{b}	0.60^{ab}
tyrosine	0.00^{e}	0.07^{a}	0.03^{c}	0.02^{d}	0.03^{b}
valine	0.28^{b}	0.33^{a}	0.31^{a}	0.27^{b}	0.24^{c}
**TOAs**, including:	3.57^{bc}	3.74^{a}	3.68^{ab}	3.44^{c}	3.24^{d}
butyric acid	0.19^{b}	0.23^{a}	0.12^{c}	0.19^{b}	0.17^{b}
citric acid	0.91^{a}	0.68^{d}	0.91^{a}	0.74^{c}	0.79^{b}
lactic acid	0.25^{a}	0.30^{a}	0.24^{a}	0.28^{a}	0.27^{a}
malic acid	1.71^{b}	1.77^{ab}	1.84^{a}	1.77^{ab}	1.34^{c}
malonic acid	0.12^{bc}	0.14^{a}	0.13^{ab}	0.10^{c}	0.12^{bc}
oxalic acid	0.17^{c}	0.20^{a}	0.18^{b}	0.14^{d}	0.16^{c}
succinic acid	0.23^{d}	0.42^{a}	0.26^{c}	0.21^{d}	0.38^{b}
**TRCs**, including:	7.98^{a}	6.99^{c}	7.55^{b}	6.53^{d}	6.57^{d}
phosphoric acid	7.77^{a}	6.69^{c}	7.31^{b}	6.31^{d}	6.40^{cd}
urea	0.21^{c}	0.30^{a}	0.24^{b}	0.22^{bc}	0.17^{d}

Means of 3 replicates. The same superscript letters by the values indicate statistically insignificant differences ($P<0.05$) based on ANOVA analysis and Tukey's post- hoc corrections (valid in rows).

Table S3. The concentration of total identified polar metabolites (TIPMs), including total soluble carbohydrates (TSCs), total amino acids (TAAs), total organic acids (TOAs), and total remaining compounds (TRCs) **in shoots** of 22-day-old pea seedlings (*Pisum sativum* L.), 14 days post-inoculation with *D. pinodes*.

	Control	*D. pinodes* infection			
		Water	Fungicide	bio-Ag NPs	
				100 mg/L	200 mg/L
**Metabolites**	mg/g DW				
**TIPMs**, including:	121.26^{a}	82.87^{b}	80.22^{b}	80.25^{b}	80.84^{b}
**TSCs**, including:	47.41^{a}	20.46^{b}	18.00^{c}	18.77^{c}	19.01^{bc}
fructose	0.22^{b}	0.64^{a}	0.26^{b}	0.23^{b}	0.18^{b}
galactose	1.23^{a}	0.62^{b}	0.49^{c}	0.62^{b}	0.56^{bc}
glucose	1.73^{a}	1.77^{a}	1.70^{ab}	1.26^{c}	1.36^{bc}
myo-inositol	1.88^{a}	1.36^{bc}	1.28^{c}	1.43^{b}	1.42^{b}
sucrose	41.63^{a}	15.29^{b}	13.64^{b}	14.40^{b}	14.84^{b}
gluconic acid	0.74^{b}	0.78^{ab}	0.63^{c}	0.83^{a}	0.63^{c}
**TAAs**, including:	59.63^{a}	44.82^{b}	46.27^{b}	45.21^{b}	45.48^{b}
alanine	0.66^{bc}	0.77^{b}	0.59^{c}	0.71^{bc}	0.95^{a}
asparagine	10.43^{a}	2.95^{c}	7.62^{b}	6.42^{b}	6.00^{b}
aspartic acid	1.10^{c}	2.38^{a}	1.85^{b}	2.12^{ab}	2.16^{a}
β-alanine	0.13^{a}	0.08^{b}	0.15^{a}	0.15^{a}	0.15^{a}
GABA	1.08^{c}	1.86^{a}	1.47^{b}	1.64^{ab}	1.54^{ab}
glutamic acid	1.53^{a}	1.26^{abc}	1.17^{c}	1.19^{bc}	1.48^{ab}
homoserine	34.90^{a}	30.09^{ab}	28.62^{b}	27.38^{b}	26.95^{b}
hydroxyproline	0.97^{a}	0.43^{b}	0.43^{b}	0.54^{b}	0.56^{b}
isoleucine	0.68^{ab}	0.69^{a}	0.55^{c}	0.57^{bc}	0.65^{abc}
lysine	0.16^{a}	0.00^{c}	0.17^{a}	0.12^{b}	0.14^{ab}
phenylalanine	0.55^{a}	0.44^{b}	0.41^{b}	0.42^{b}	0.52^{a}
proline	3.05^{a}	1.10^{b}	0.41^{c}	1.08^{b}	1.22^{b}
serine	0.92^{a}	0.43^{c}	0.51^{bc}	0.58^{b}	0.46^{c}
threonine	1.79^{a}	0.89^{c}	1.12^{bc}	0.95^{c}	1.27^{b}
tyrosine	0.12^{b}	0.19^{ab}	0.20^{ab}	0.20^{ab}	0.24^{a}
valine	1.55^{a}	1.26^{b}	1.00^{c}	1.14^{b}	1.18^{b}
**TOAs**, including:	3.54^{d}	4.32^{b}	3.91^{c}	3.94^{c}	5.06^{a}
butyric acid	0.62^{a}	0.53^{c}	0.38^{e}	0.48^{d}	0.57^{b}
citric acid	1.10^{c}	1.04^{c}	1.35^{a}	1.26^{b}	1.19^{b}
lactic acid	0.09^{b}	0.32^{a}	0.17^{b}	0.12^{b}	0.33^{a}
malic acid	0.76^{c}	1.02^{a}	0.76^{c}	0.91^{b}	0.73^{c}
malonic acid	0.20^{a}	0.16^{b}	0.10^{c}	0.15^{b}	0.16^{b}
oxalic acid	0.20^{b}	0.36^{a}	0.24^{b}	0.24^{b}	0.26^{b}
succinic acid	0.57^{d}	0.90^{b}	0.91^{b}	0.78^{c}	1.81^{a}
**TRCs** (phosphoric acid)	10.68^{d}	13.25^{a}	12.03^{b}	12.34^{b}	11.29^{c}

Means of 3 replicates. The same superscript letters by the values indicate statistically insignificant differences ($P<0.05$) based on ANOVA analysis and Tukey's post- hoc corrections (valid in rows).

Table S4. The concentration of total identified polar metabolites (TIPMs), including total soluble carbohydrates (TSCs), total amino acids (TAAs), total organic acids (TOAs), and total remaining compounds (TRCs) **in cotyledons** of 22-day-old pea seedlings (*Pisum sativum* L.), 14 days post-inoculation with *D. pinodes*.

	Control	*D. pinodes* infection			
		Water	Fungicide	bio-Ag NPs	
				100 mg/L	200 mg/L
**Metabolites**	mg/g DW				
**TIPMs**, including:	189.84^{ab}	186.23^{bc}	201.64^{a}	174.92^{c}	184.09^{bc}
**TSCs**, including:	141.03^{ab}	141.28^{ab}	146.83^{a}	130.04^{b}	141.91^{ab}
fructose	0.78^{d}	2.51^{a}	0.97^{d}	1.67^{b}	1.23^{c}
galactose	0.26^{ab}	0.33^{a}	0.14^{c}	0.29^{ab}	0.23^{b}
glucose	2.08^{cd}	2.79^{a}	1.97^{d}	2.43^{b}	2.39^{bc}
myo-inositol	3.53^{bc}	3.67^{ab}	3.69^{ab}	3.35^{c}	3.83^{a}
sucrose	134.11^{ab}	131.25^{ab}	139.82^{a}	121.16^{b}	133.80^{ab}
gluconic acid	0.28^{d}	0.73^{b}	0.25^{d}	1.14^{a}	0.43^{c}
**TAAs**, including:	39.05^{b}	34.10^{c}	44.26^{a}	35.22^{c}	33.26^{c}
alanine	1.01^{d}	1.41^{b}	1.62^{a}	1.54^{a}	1.17^{c}
asparagine	7.21^{b}	3.50^{e}	9.34^{a}	5.67^{d}	6.42^{c}
aspartic acid	0.99^{b}	0.89^{b}	1.21^{a}	1.00^{b}	0.92^{b}
β-alanine	0.07^{b}	0.09^{a}	0.10^{a}	0.10^{a}	0.09^{ab}
GABA	1.83^{e}	3.32^{a}	2.65^{c}	3.08^{b}	2.22^{d}
glutamic acid	0.67^{a}	0.14^{c}	0.58^{ab}	0.24^{c}	0.48^{b}
homoserine	14.72^{a}	12.39^{b}	13.54^{ab}	10.72^{c}	9.90^{c}
hydroxyproline	0.46^{a}	0.56^{a}	0.47^{a}	0.39^{a}	0.40^{a}
isoleucine	1.39^{c}	1.55^{b}	1.72^{a}	1.55^{b}	1.43^{c}
lysine	0.35^{c}	0.27^{c}	0.66^{a}	0.49^{b}	0.60^{a}
phenylalanine	2.72^{b}	2.31^{c}	3.24^{a}	2.42^{c}	2.45^{c}
proline	0.91^{a}	0.87^{a}	0.61^{b}	0.84^{a}	0.28^{c}
serine	2.02^{c}	2.12^{bc}	2.72^{a}	2.17^{b}	2.05^{c}
threonine	0.74^{b}	0.94^{a}	0.99^{a}	0.90^{a}	0.76^{b}
tyrosine	0.51^{b}	0.29^{c}	0.85^{a}	0.59^{b}	0.85^{a}
valine	3.45^{b}	3.43^{b}	3.96^{a}	3.49^{b}	3.24^{c}
**TOAs**, including:	2.69^{b}	3.40^{a}	3.43^{a}	3.51^{a}	2.78^{b}
butyric acid	0.05^{a}	0.05^{a}	0.05^{a}	0.05^{a}	0.03^{a}
citric acid	1.31^{b}	0.95^{c}	1.57^{a}	1.27^{b}	1.24^{b}
lactic acid	0.06^{b}	0.19^{a}	0.09^{b}	0.12^{b}	0.06^{b}
malic acid	0.51^{b}	0.45^{c}	0.60^{a}	0.52^{b}	0.45^{c}
malonic acid	0.03^{b}	0.04^{a}	0.04^{b}	0.04^{a}	0.04^{b}
oxalic acid	0.14^{a}	0.14^{a}	0.15^{a}	0.14^{a}	0.13^{a}
succinic acid	0.59^{e}	1.57^{a}	0.94^{c}	1.37^{b}	0.83^{d}
**TRCs**, including:	7.06^{b}	7.45^{a}	7.11^{b}	6.15^{c}	6.15^{c}
phosphoric acid	7.02^{b}	7.42^{a}	7.05^{b}	6.12^{c}	6.12^{c}
urea	0.04^{b}	0.03^{c}	0.06^{a}	0.03^{c}	0.03^{c}

Means of 3 replicates. The same superscript letters by the values indicate statistically insignificant differences ($P<0.05$) based on ANOVA analysis and Tukey's post- hoc corrections (valid in rows).

Table S5. The concentration of total identified polar metabolites (TIPMs), including total soluble carbohydrates (TSCs), total amino acids (TAAs), total organic acids (TOAs), and total remaining compounds (TRCs) **in roots** of 22-day-old pea seedlings (*Pisum sativum* L.), 14 days post-inoculation with *F. avenaceum*.

	Control	*F. avenaceum* infection			
		Water	Fungicide	bio-Ag NPs	
				100 mg/L	200 mg/L
**Metabolites**	mg/g DW				
**TIPMs**, including:	59.52^{c}	64.05^{b}	72.73^{a}	73.31^{a}	71.45^{a}
**TSCs**, including:	14.65^{b}	12.84^{c}	13.63^{bc}	14.80^{b}	17.11^{a}
fructose	0.08^{c}	0.12^{ab}	0.07^{c}	0.10^{bc}	0.13^{a}
galactose	0.11^{bc}	0.08^{c}	0.09^{c}	0.19^{ab}	0.25^{a}
glucose	1.75^{ab}	0.99^{b}	0.51^{c}	1.88^{a}	2.12^{a}
myo-inositol	1.61^{a}	1.19^{d}	1.33^{c}	1.46^{b}	1.49^{b}
sucrose	10.83^{bc}	10.18^{c}	11.44^{b}	10.87^{bc}	12.87^{a}
gluconic acid	0.26^{b}	0.27^{ab}	0.19^{c}	0.29^{a}	0.25^{b}
**TAAs**, including:	36.28^{c}	41.36^{b}	48.75^{a}	48.21^{a}	43.96^{b}
alanine	0.49^{b}	0.72^{a}	0.71^{a}	0.51^{b}	0.31^{c}
asparagine	3.95^{c}	4.48^{c}	4.90^{bc}	6.34^{a}	6.16^{ab}
aspartic acid	0.50^{c}	0.63^{b}	0.78^{a}	0.54^{bc}	0.46^{c}
β-alanine	0.03^{c}	0.05^{a}	0.04^{ab}	0.04^{b}	0.02^{c}
GABA	0.53^{d}	0.98^{b}	0.69^{c}	1.08^{a}	0.93^{b}
glutamic acid	0.37^{c}	0.63^{a}	0.48^{b}	0.48^{b}	0.52^{b}
homoserine	28.44^{c}	30.94^{bc}	38.04^{a}	36.70^{a}	33.16^{b}
hydroxyproline	0.22^{ab}	0.22^{ab}	0.20^{b}	0.26^{ab}	0.31^{a}
isoleucine	0.22^{a}	0.27^{a}	0.26^{a}	0.27^{a}	0.22^{a}
lysine	0.00^{c}	0.15^{ab}	0.14^{b}	0.16^{ab}	0.20^{a}
phenylalanine	0.16^{c}	0.18^{bc}	0.19^{bc}	0.23^{a}	0.21^{ab}
proline	0.03^{c}	0.26^{b}	0.32^{a}	0.04^{c}	0.05^{c}
serine	0.48^{b}	0.69^{a}	0.76^{a}	0.50^{b}	0.47^{b}
threonine	0.64^{cd}	0.77^{ab}	0.85^{a}	0.71^{bc}	0.60^{d}
tyrosine	0.00^{c}	0.05^{ab}	0.03^{b}	0.05^{ab}	0.06^{a}
valine	0.24^{d}	0.33^{ab}	0.35^{a}	0.30^{bc}	0.28^{c}
**TOAs**, including:	2.86^{c}	4.08^{a}	4.20^{a}	3.40^{b}	3.03^{c}
butyric acid	0.17^{c}	0.23^{ab}	0.25^{a}	0.21^{bc}	0.23^{ab}
citric acid	0.52^{c}	0.61^{b}	0.68^{a}	0.57^{bc}	0.57^{bc}
lactic acid	0.46^{a}	0.30^{b}	0.30^{b}	0.45^{a}	0.41^{a}
malic acid	1.31^{c}	2.41^{a}	2.43^{a}	1.76^{b}	1.47^{c}
malonic acid	0.08^{b}	0.16^{a}	0.16^{a}	0.07^{b}	0.05^{c}
oxalic acid	0.16^{a}	0.17^{a}	0.15^{a}	0.16^{a}	0.17^{a}
succinic acid	0.17^{bc}	0.21^{a}	0.22^{a}	0.18^{b}	0.15^{c}
**TRCs**, including:	5.72^{c}	5.78^{c}	6.15^{c}	6.90^{b}	7.34^{a}
phosphoric acid	5.49^{d}	5.60^{cd}	5.96^{c}	6.64^{b}	7.11^{a}
urea	0.24^{ab}	0.18^{c}	0.19^{c}	0.26^{a}	0.23^{b}

Means of 3 replicates. The same superscript letters by the values indicate statistically insignificant differences ($P<0.05$) based on ANOVA analysis and Tukey's post- hoc corrections (valid in rows).

Table S6. The concentration of total identified polar metabolites (TIPMs), including total soluble carbohydrates (TSCs), total amino acids (TAAs), total organic acids (TOAs), and total remaining compounds (TRCs) **in shoots** of 22-day-old pea seedlings (*Pisum sativum* L.), 14 days post-inoculation with *F. avenaceum*.

	Control	*F. avenaceum* infection			
		Water	Fungicide	bio-Ag NPs	
				100 mg/L	200 mg/L
**Metabolites**	mg/g DW				
**TIPMs**, including:	117.80^{b}	72.23^{d}	103.45^{c}	132.60^{a}	119.60^{b}
**TSCs**, including:	47.72^{a}	29.64^{c}	27.86^{c}	46.12^{a}	38.52^{b}
fructose	0.68^{a}	0.87^{a}	0.70^{a}	0.56^{a}	0.60^{a}
galactose	0.83^{b}	0.86^{ab}	0.87^{ab}	1.53^{a}	1.04^{ab}
glucose	2.82^{a}	3.36^{a}	4.62^{a}	4.99^{a}	3.77^{a}
myo-inositol	2.22^{a}	1.82^{c}	1.83^{bc}	1.98^{b}	1.93^{bc}
sucrose	40.47^{a}	21.62^{d}	18.75^{e}	36.60^{b}	30.67^{c}
gluconic acid	0.70^{b}	1.10^{a}	1.08^{a}	0.45^{c}	0.51^{c}
**TAAs**, including:	58.82^{c}	30.31^{d}	61.82^{bc}	73.94^{a}	68.74^{ab}
alanine	0.72^{a}	0.54^{cd}	0.49^{d}	0.57^{bc}	0.61^{b}
asparagine	20.34^{c}	6.86^{d}	22.61^{bc}	31.78^{a}	27.68^{ab}
aspartic acid	1.21^{c}	1.60^{b}	2.08^{a}	1.15^{c}	0.95^{d}
β-alanine	0.11^{b}	0.04^{c}	0.05^{c}	0.10^{b}	0.12^{a}
GABA	0.99^{c}	0.92^{c}	0.87^{c}	1.34^{a}	1.16^{b}
glutamic acid	1.40^{a}	0.54^{b}	1.31^{a}	1.42^{a}	1.43^{a}
homoserine	23.40^{b}	14.31^{c}	27.51^{a}	26.95^{a}	27.52^{a}
hydroxyproline	1.35^{a}	1.04^{ab}	0.49^{c}	1.10^{ab}	0.88^{bc}
isoleucine	0.49^{b}	0.62^{a}	0.52^{b}	0.64^{a}	0.64^{a}
lysine	0.67^{c}	0.12^{d}	0.86^{bc}	1.35^{a}	1.25^{ab}
phenylalanine	0.48^{b}	0.18^{d}	0.39^{c}	0.60^{a}	0.54^{ab}
proline	4.46^{a}	1.11^{e}	1.59^{d}	2.96^{b}	2.34^{c}
serine	0.85^{b}	0.75^{c}	0.85^{b}	0.97^{a}	0.90^{ab}
threonine	0.84^{bc}	0.59^{c}	0.90^{b}	1.22^{a}	0.99^{ab}
tyrosine	0.21^{ab}	0.09^{c}	0.14^{bc}	0.23^{a}	0.22^{a}
valine	1.32^{c}	1.02^{e}	1.16^{d}	1.56^{a}	1.48^{b}
**TOAs**, including:	3.12^{b}	3.08^{b}	3.68^{a}	3.12^{b}	3.25^{b}
butyric acid	0.15^{e}	0.24^{c}	0.40^{b}	0.18^{d}	0.49^{a}
citric acid	1.30^{ab}	1.17^{b}	1.43^{a}	1.32^{ab}	1.20^{b}
lactic acid	0.12^{a}	0.11^{a}	0.07^{a}	0.11^{a}	0.14^{a}
malic acid	0.93^{b}	0.86^{c}	1.08^{a}	0.94^{b}	0.71^{d}
malonic acid	0.20^{a}	0.24^{a}	0.13^{a}	0.14^{a}	0.15^{a}
oxalic acid	0.13^{c}	0.14^{bc}	0.18^{ab}	0.15^{bc}	0.19^{a}
succinic acid	0.30^{b}	0.31^{b}	0.39^{a}	0.29^{b}	0.37^{a}
**TRCs** (phosphoric acid)	8.13^{c}	9.20^{b}	10.10^{a}	9.42^{b}	9.10^{b}

Means of 3 replicates. The same superscript letters by the values indicate statistically insignificant differences ($P<0.05$) based on ANOVA analysis and Tukey's post- hoc corrections (valid in rows).

Table S7. The concentration of total identified polar metabolites (TIPMs), including total soluble carbohydrates (TSCs), total amino acids (TAAs), total organic acids (TOAs), and total remaining compounds (TRCs) **in cotyledons** of 22-day-old pea seedlings (*Pisum sativum* L.), 14 days post-inoculation with *F. avenaceum*.

	Control	*F. avenaceum* infection			
		Water	Fungicide	bio-Ag NPs	
				100 mg/L	200 mg/L
**Metabolites**	mg/g DW				
**TIPMs**, including:	157.92^{cd}	148.66^{d}	165.31^{bc}	180.52^{a}	174.42^{ab}
**TSCs**, including:	120.41^{b}	108.25^{c}	121.10^{b}	132.57^{a}	129.60^{ab}
fructose	1.53^{b}	4.18^{a}	1.89^{b}	0.55^{c}	2.00^{b}
galactose	0.94^{ab}	1.14^{a}	0.75^{bc}	0.26^{d}	0.56^{c}
glucose	4.34^{c}	7.14^{a}	5.52^{bc}	2.44^{d}	5.82^{ab}
myo-inositol	3.45^{c}	4.00^{a}	3.85^{ab}	3.71^{b}	3.89^{ab}
sucrose	109.97^{bc}	91.18^{d}	108.55^{c}	125.44^{a}	116.96^{b}
gluconic acid	0.18^{d}	0.61^{a}	0.54^{b}	0.18^{d}	0.35^{c}
**TAAs**, including:	28.97^{c}	30.72^{c}	34.03^{b}	38.10^{a}	34.84^{ab}
alanine	0.75^{c}	0.99^{b}	1.07^{ab}	1.12^{a}	1.11^{a}
asparagine	6.28^{c}	6.31^{c}	8.10^{b}	10.08^{a}	8.48^{b}
aspartic acid	0.71^{bc}	0.75^{bc}	0.68^{c}	0.84^{a}	0.77^{ab}
β-alanine	0.06^{c}	0.08^{b}	0.11^{a}	0.11^{a}	0.09^{b}
GABA	1.60^{d}	2.35^{b}	2.47^{a}	2.10^{c}	2.20^{c}
glutamic acid	0.33^{c}	0.20^{e}	0.24^{d}	0.66^{a}	0.40^{b}
homoserine	8.64^{b}	7.16^{c}	8.30^{bc}	10.19^{a}	8.89^{ab}
hydroxyproline	0.21^{b}	0.28^{ab}	0.34^{a}	0.30^{ab}	0.31^{ab}
isoleucine	1.26^{c}	1.59^{a}	1.35^{bc}	1.30^{bc}	1.39^{b}
lysine	0.49^{c}	0.87^{a}	0.75^{ab}	0.68^{b}	0.89^{a}
phenylalanine	2.23^{c}	2.60^{ab}	2.43^{bc}	2.71^{a}	2.53^{ab}
proline	0.44^{b}	0.26^{c}	0.69^{a}	0.53^{b}	0.79^{a}
serine	1.60^{c}	1.83^{b}	2.22^{a}	2.18^{a}	1.95^{b}
threonine	0.72^{c}	0.98^{a}	0.93^{ab}	0.77^{bc}	0.82^{abc}
tyrosine	0.86^{c}	1.32^{ab}	1.29^{ab}	1.39^{a}	1.18^{b}
valine	2.76^{b}	3.15^{a}	3.08^{a}	3.16^{a}	3.03^{a}
**TOAs**, including:	2.89^{c}	3.44^{a}	3.05^{bc}	3.25^{ab}	3.26^{ab}
butyric acid	0.05^{bc}	0.04^{c}	0.06^{ab}	0.08^{a}	0.04^{bc}
citric acid	1.43^{bc}	1.57^{b}	1.33^{c}	1.75^{a}	1.58^{b}
lactic acid	0.05^{a}	0.07^{a}	0.10^{a}	0.05^{a}	0.07^{a}
malic acid	0.55^{a}	0.55^{a}	0.48^{b}	0.48^{b}	0.55^{a}
malonic acid	0.03^{b}	0.06^{a}	0.04^{b}	0.03^{b}	0.04^{b}
oxalic acid	0.13^{a}	0.13^{a}	0.13^{a}	0.14^{a}	0.14^{a}
succinic acid	0.64^{e}	1.02^{a}	0.92^{b}	0.71^{d}	0.84^{c}
**TRCs**, including:	5.65^{c}	6.25^{b}	7.13^{a}	6.59^{b}	6.71^{ab}
phosphoric acid	5.63^{c}	6.22^{b}	7.10^{a}	6.57^{b}	6.68^{ab}
urea	0.03^{a}	0.02^{a}	0.03^{a}	0.03^{a}	0.03^{a}

Means of 3 replicates. The same superscript letters by the values indicate statistically insignificant differences ($P<0.05$) based on ANOVA analysis and Tukey's post- hoc corrections (valid in rows).

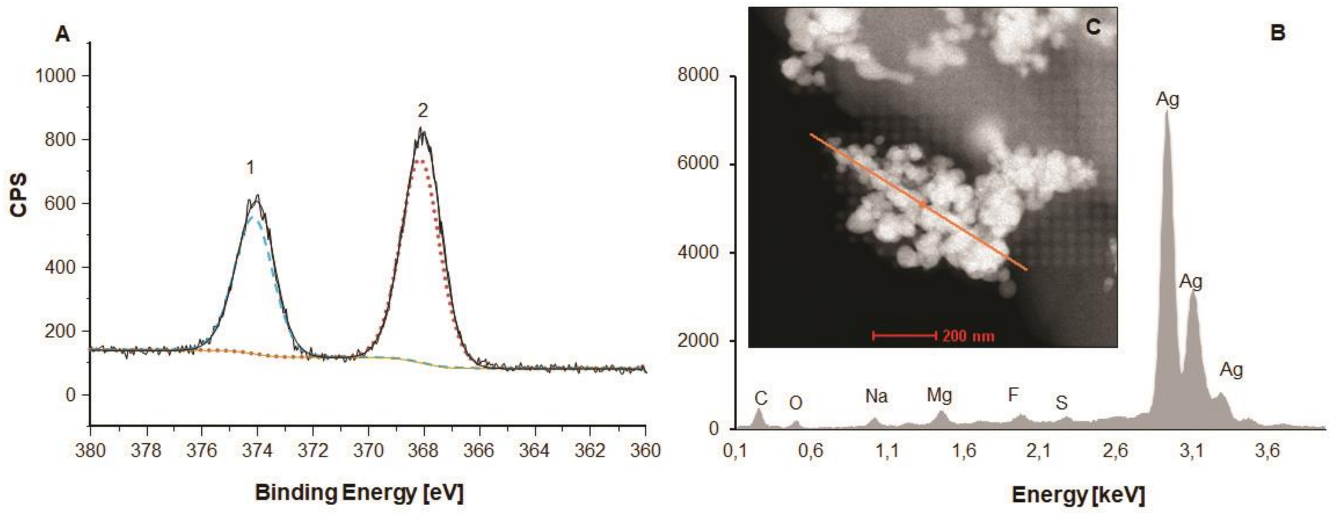

Figure S2. Characterization of bio-synthesized silver nanoparticles (bio-AgNPs) (A) X-ray Photoelectron Spectroscopy (XPS) wide-scan spectrum showcasing the surface chemical composition of bio-AgNPs. This spectrum displays prominent silver peaks at binding energies characteristic of Ag($3d_{5/2}$) at 368.1 eV and Ag($3d_{3/2}$) at 374.1 eV, with a full width at half maximum (FWHM) of 1.5 eV and an energy separation (ΔE) of 6 eV, indicating the presence and purity of silver in the nanoparticles; (B) Energy Dispersive X-ray (EDX) spectra, providing elemental composition analysis which confirms the presence of elemental silver as a major constituent along with minor traces of other elements used in the synthesis process; (C) Transmission Electron Microscopy (TEM) images in darkfield mode, revealing the morphology and size distribution of the bio-AgNPs.
